# Xeno-Free Human Wharton’s Jelly Mesenchymal Stromal Cells Maintain Their Characteristic Properties after Long-Term Cryopreservation

**DOI:** 10.22074/cellj.2021.7131

**Published:** 2021-05-26

**Authors:** Caroline Mathen, Wilfrid Dsouza

**Affiliations:** OCT Therapies and Research, Pvt. Ltd., Mumbai, India

**Keywords:** Mesenchymal Stromal Cells, Stability, Umbilical Cord, Wharton’s Jelly

## Abstract

**Objective:**

The past decade has witnessed a rapid growth in harnessing the potential of adult stem cells for regenerative medicine. An investigational new drug (IND) or a regenerative medicine advanced therapy (RMAT) product must fulfil many requirements, such as stability studies, after cryopreservation. Such studies are important to ascertain the utility of off-the-shelf allogeneic cells for clinical applications. The present work describes a complete characterisation of xeno- free human Wharton’s Jelly mesenchymal stromal cells (hWJ-MSCs) before and up to 28 months post-cryopreservation.

**Materials and Methods:**

In this experimental study, culture methods that involved plasma derived human serum and recombinant trypsin were used to develop clinical grade cells. Complete cell characterisation involved flow cytometry studies for viability, positive and negative markers, colony forming unit (CFU) potential, population doubling time (PDT), soft agar assay to evaluate *in vitro* tumourigenicity, karyotype analysis and differentiation studies which were performed before and at 6, 12, 18 and 28 months post-cryopreservation.

**Results:**

Our data showed consistency in the flow cytometry, CFU assay, PDT, soft agar assay, karyotyping and differentiation studies.

**Conclusion:**

Using our protocols for extended xeno-free culture and cryopreservation of hWJ-MSCs, we could establish the shelf life of the cell-based product for up to 28 months.

## Introduction

Mesenchymal stromal cells (MSCs) are adult multipotent cells characterized by self-renewal as well as differentiation into various fibroblastic lineages, including transdifferentiation into other lineages. These cells were initially studied by Owen and Friedenstein (1) who recognized their differentiation potential across various lineages. Although these cells are implicated in various intrinsic healing mechanisms, the process may be slow, absent or compromised in certain clinical conditions. The human body retains contingent reserves of stem cells in various organs to replace diseased or damaged tissues when needed. Since this process is time-consuming *in vivo*, these cells can be expanded *in vitro* in the laboratory and be used to facilitate the body’s innate self-healing mechanisms. More than two decades ago, McElreavey et al. (2) isolated MSCs from the umbilical cord matrix, also known as Wharton’s jelly (WJ), and these cells have been well-studied (3-5). Umbilical cord represents an abundant, young, non-invasive and non-controversial tissue source without any ethical implications if carried out within the confines of Institutional Ethics approval and accompanied by documented donor informed consent. Interestingly, apart from their differentiation capacities, MSCs also have inherent immunomodulatory properties (6), while being hypoimmune cells themselves. These cells are negative for class II major histocompatibility markers (7). Thus, human MSCs are non- antigenic and indiscernible to the recipient’s immune system (8, 9).

Caplan (10) theorizes that perhaps the most vital role of MSCs is to evade immunosurveillance and promote a microenvironment that supports regeneration. Various groups that have used laboratory expanded bone marrow-derived MSCs in clinical settings, irrespective of autologous or allogeneic sources, have not reported any adverse events. This proves that isolation and *in vitro* expansion is safe and translates to clinical benefit following intravenous delivery of human MSCs (11). Apart from healing and regenerative properties for various indications (12, 13), MSCs have also been implicated in immune-modulated remediation for graft versus host disease (GvHD) and type one diabetes (14, 15).

Stem cells from adults, foetal and other sources are widely used to regenerate tissues in humans after they have suffered damages due to diseases or injuries. For this purpose, cells must be grown *in vitro* for different periods of time using defined media, an important component of which is animal serum. Fetal bovine serum (FBS) or calf serum, a derivative of the meat industry, is a commonly used additive in cell culture as it contains factors that the cells need for attachment, proliferation and differentiation (16). For clinical applications, cells are cultured in media that contain animal sera, human allogeneic serum or a cocktail of growth factors from xeno (animal) or recombinant sources. Nevertheless, there are scientific, safety and ethical issues regarding the use of animal serum (17). Additionally, when the aim is cell-based therapeutics or clinical applications, there are limitations and risks while using these methods, given the chance for transmission of pathogens (18) or prions (19) from animal sources to the cells that may eventually end up in humans during transplantation. Consequently, during translational research, it is preferable to have a cell-based product, which is free from any animal products or xeno-free. In this study, we have analysed xeno-free human WJ-MSCs (hWJ-MSCs) at different time points over 28 months after cryopreservation.

## Materials and Methods

In this experimental study, human umbilical cords from elective caesarean deliveries were collected after obtaining written informed consent from the potential donors and relevant Ethics approvals in compliance with the National Guidelines for Stem Cell Research (NGSCR). Approvals were obtained from the Institutional Committee for Stem Cell Research of the Company (ICSCR/OCT/UID/002) and Hospital (ISCC/04/14) as well as the hospital’s Institutional Ethics Committee (IEC/38/14).

All reagents were procured from Sigma (UK) or Gibco (Life Technologies, Denmark). Other chemicals were from Qualigens and were of analytical grade. Differentiation media, alizarin red S, oil red O and alcian blue stains were from HiMedia (India). Consumables were either from Nunc Thermo Fisher Scientific or Tarsons. The culture media used to isolate MSCs was Dulbecco’s modified eagle medium (DMEM) supplemented with 1 mM sodium pyruvate, 4 mM L-glutamine, 10% human serum, 1% penicillin/streptomycin and 2.5 mg/mL amphotericin B. The human serum was recovered from plasma using modified protocols as described elsewhere (20). Briefly, pooled plasma lots were treated with 9% of 0.1 M calcium chloride, allowed to clot, and the recovered serum was heat inactivated at 56˚C for 30 minutes. The serum was further processed to neutralise viruses, bacteria and mycoplasma (21). Cold sterilization was carried out using 0.1% peracetic acid. 

Human umbilical cords were collected after a three-tier donor-screening protocol, which consisted of documented informed consent, medical history and infectious disease screening that included, but was not limited to: human immunodeficiency viruses (HIV), syphilis, hepatitis B and hepatitis C as per the NGSCR. The umbilical cords were coded to protect donor identities and were processed according to good manufacturing practice (GMP) compliant conditions. 

Isolation of the hWJ-MSCs was carried out as previously described (22) with some modifications. Briefly, the donor tissue was transported to the laboratory tissue processing facility under cold conditions (<20˚C) in cold Dulbecco’s phosphate buffered saline (DPBS), supplemented with 10X amphotericin B and penicillin-streptomycin using previously validated protocols. The tissue was quarantined at 2-8˚C and processed within 48 hours. Each umbilical cord was cut into 5 cm pieces. The tissue sample was washed 2-3 times with DPBS supplemented with 2X antibiotic. The cord was cut lengthwise and the blood vessels excised. The remaining soft tissue was cut into 4-8 mm pieces and plated in 90 mm petri dishes. The petri dishes were incubated at 37˚C and 5% CO_2_ . Media change was carried out every three days until the MSCs were 80% confluent. Cells were subcultured using TrypLE™ Select and further culturing was carried out in T-75 or triple flasks up to the second passage, which comprised the master cell bank and up to the sixth passage, which comprised the working cell bank. Cells were cryopreserved in 90% serum and 10% dimethyl sulphoxide (DMSO) using a frosty in a -80˚C freezer overnight, after which they were transferred to liquid nitrogen for extended storage.

Passage 2 (P-2) and passage 6 (P-6) MSCs from five lots (5 donors) were subjected to the following characterisation and analyses pre- and post-cryopreservation at 0, 6, 12, 18 and 28 months. Characterisation was carried out in accordance with the International Society for Cellular Therapy (ISCT) guidelines (23) and included the following:

### Morphological evaluation

The cells were observed under an inverted microscope for confirmation of fibroblastic morphology and adherence to plastic. 

### Flow cytometry

This analysis was carried out to confirm that the cells were MSCs. All antibodies were procured from Biolegend. 0.5×10^6^ cells of each sample were used for flow cytometry. Viability was assessed using Zombie Violet™ dye (0.092%, cat. no. 423113). The cells were analysed for FITC conjugated CD90 (0.5% concentration, cat. no. 328107), PE conjugated CD105 (0.5% concentration, cat. no. 323205) and PerCP conjugated CD34 (0.2% concentration, cat. no. 343519), CD45 (0.2% concentration, cat. no. 304025) and HLADR (0.3% concentration, cat. no. 307627). Onecomp EBeads (EBioscience cat. no. 01-1111-41) were used to prepare single colour controls for the fluorescent labelled antibodies and the cells were used to prepare a single colour control for Zombie Violet. At least 20000 events were recorded on an Attune Acoustic flow cytometer (Thermo Fisher) and FlowJo software v7.6.5 was used for data analysis. One sample of P-6 at 12 months was lost; however, this did not affect the overall analysis. 

### Colony forming unit 

For this assay, cells were seeded at a concentration of 1×10^4^ in 60 mm petri dishes for approximately 10 days and terminated when discrete colonies that comprised at least 50 cells per colony were visible. After termination, the cells were stained with 0.4% Sulforhodamine B (SRB) in 1% acetic acid dye for visualisation (24) and counted under an inverted microscope. Each experiment was carried out in triplicate for three lots of the early and late passages and at different time points. 

### Population doubling time

A total of 8×10^3^ cells/cm^2^ were seeded and the population doubling time (PDT) was calculated as per the formula recommended by the American Type Culture Collection

(ATCC):

DT=T ln2/ln(Xe/Xb)

Where:

T is the incubation time in hours.

Xb is the cell number at the beginning of the incubation time.

Xe is the cell number at the end of the incubation time.

The experiments were carried out for five lots from passages 2 to 6.

### Soft agar assay

This is an *in vitro* tumorigenic assay. A 2% base of agar mixed with 2X medium (final concentration: 1% agar and 1X medium) was plated onto 60 mm petri dishes followed by 1% agar mixed with 2X medium that contained a 1×10^5^ cell suspension (final concentration: 0.5% agar and 1X medium). A positive control was concurrently run using MCF7, a breast cancer cell line. Growth was observed over 21 days. The experiments were carried out in duplicate for each passage and time points for all lots.

### Karyotype analysis

This was carried out according to a modified protocol described by Moorehead et al. (25). In brief, cells were arrested in the log phase of growth by the addition of 1×10^7^ M colchicine (final volume) and incubated for up to three hours. Cells were enzymatically dispersed, washed and given hypotonic treatment at 37˚C with 0.075 M KCl for 15 minutes. The cells were repeatedly washed with fresh, chilled Carnoy’s fixative and finally re-suspended in the same. The cells were fixed onto chilled glass slides, air-dried and stained for G-banding by trypsin with Giemsa (GTG banding). At least five spreads were captured and chromosomal analysis was carried out using Olympus microscopes BX-41 and BX-43, and Cytovision software from Leica.

### Differentiation studies

Osteogenic, chondrogenic and adipogenic differentiation studies were carried out per the manufacturer’s instructions. The experiments were carried out in triplicate and repeated twice for all five lots at passages 2 and 6 at all of the time points. Miniaturized experiments were carried out in 96-well plates. Each well was seeded with 5×10^4^ cells and allowed to grow in normal growth medium. Differentiation media was added once 70% confluency was achieved. This media change was counted as the first day of differentiation. The spent media was replaced with fresh differentiation medium every 48-72 hours for up to 18-21 days. Osteogenic differentiation was confirmed by 2% alizarin red S staining while adipogenic staining of lipid vesicles was by 0.21% oil red O staining. The spent media was discarded, the cells were washed with PBS and fixed with 4% paraformaldehyde (PFA). After further washes with distilled water for alizarin red S and with 60% isopropyl alcohol (IPA) for oil red O, further incubation was carried out in the dark. The stain was washed away and cells were visualized under the microscope. Mineralized osteoblasts appeared bright orange-red in comparison with the control cells. Cells which have undergone adipogenic differentiation have red coloured lipid vesicles, which are not visible in control cells. 

For chondrogenic differentiation, 1×10^6^ cells in a centrifuge tube that contained medium were centrifuged at 1000 rpm for 10 minutes. The supernatant was carefully discarded without disturbing the pellet. Fresh media was added to the pellet, and the centrifuge tube with a loosened lid was incubated at 37˚C in a 5% CO_2_ humidified incubator for 48 hours following which the growth medium was replaced with chondrogenic differentiation medium. The pellet was gently re-suspended and centrifuged at 1000 rpm for 10 minutes. Media change was carried out every 48 hours for 18-21 days, during which time the cells aggregated and formed spheroids. For the staining procedure, a PBS wash was given and the spheroids fixed with 1% PFA. After further washes, the spheroids were stained with 1% alcian blue for 30 minutes. Excess stain was removed by washing thrice with 0.1N HCl. Distilled water was added to neutralize the acidity. 

### Statistics

Unless otherwise mentioned, the data are written as mean ± SD. All data were subjected to two-way ANOVA using GraphPad Prism Version 6.01 for Windows (GraphPad Software, San Diego, USA) with P<0.05 considered as significant.

## Results

**Fig.1:**
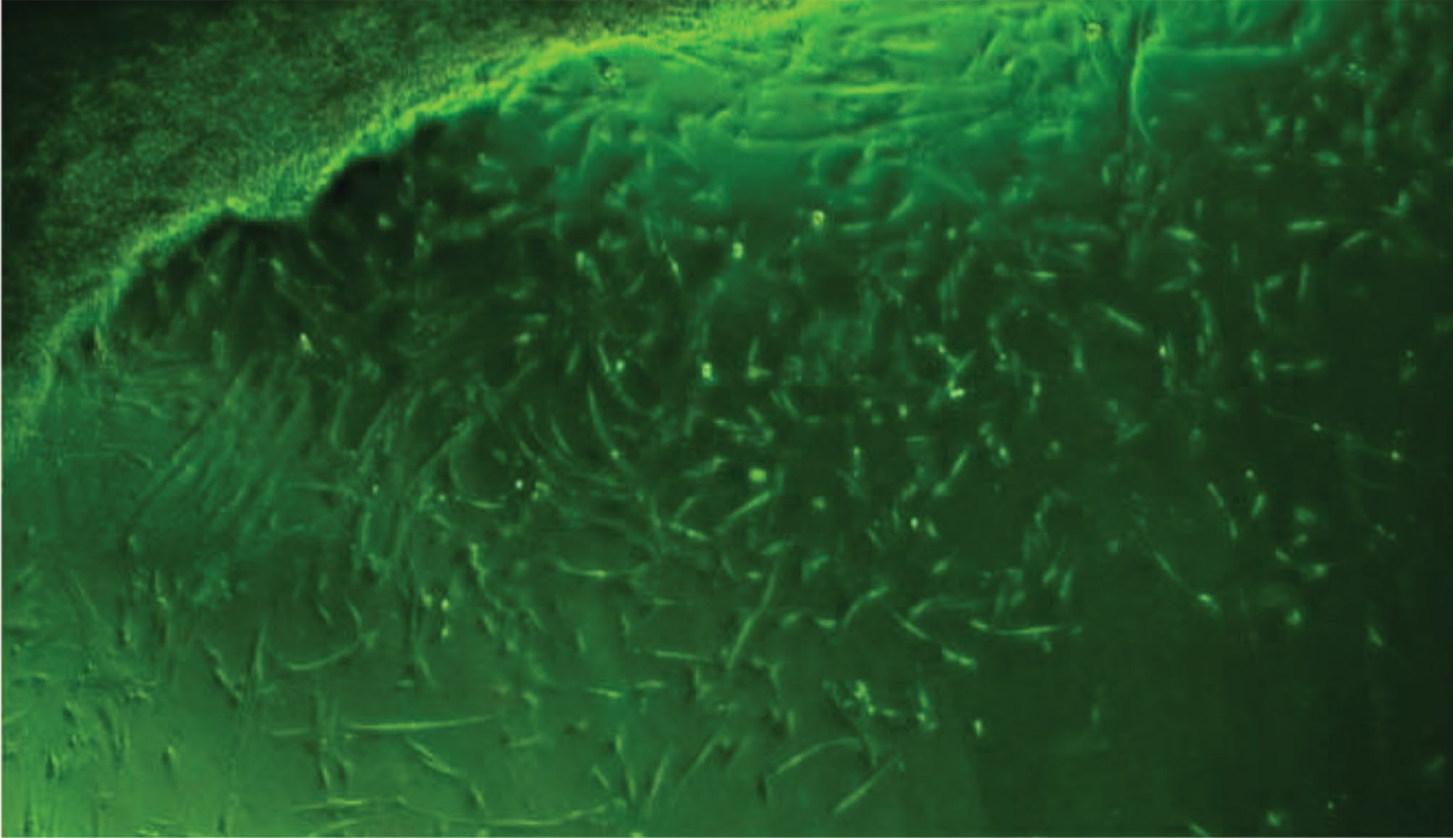
Human Wharton’s jelly mesenchymal stromal cells (hWJ-MSCs) migrating after 7-10 days of explant culture as observed under an inverted microscope (phase contrast: X4). The adherent cells appear typically fibroblastic. The first cells to migrate out of the tissue are denoted as passage-0 cells.

All cells from all of the five lots/donors exhibited normal morphology with characteristic fibroblastic, spindle shaped morphology and plastic adherent properties (Fig .1).

**Fig.2:**
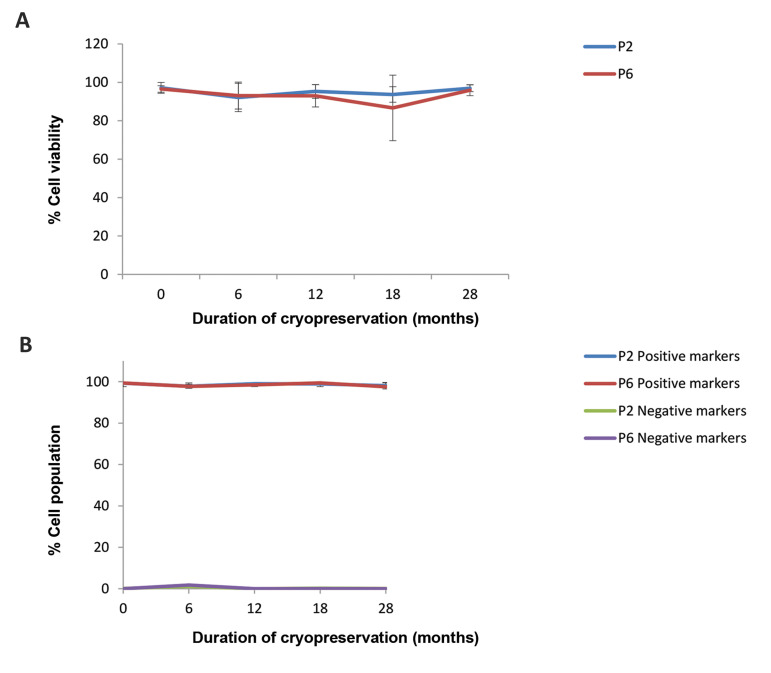
Flow cytometry studies for cell viability, and positive and negative markers. **A.** Mean cell viabilities and **B.** Haematopoietic and non-haematopoietic markers. The difference between the lots, passages and time points was not significant (data for five different lots; two-way ANOVA, P>0.05).

Flow cytometry studies for mean cell viabilities, and for positive and negative markers across different lots, passages and time points did not reveal any difference between freshly isolated and cryopreserved cells (Fig .2A, B). 

**Fig.3:**
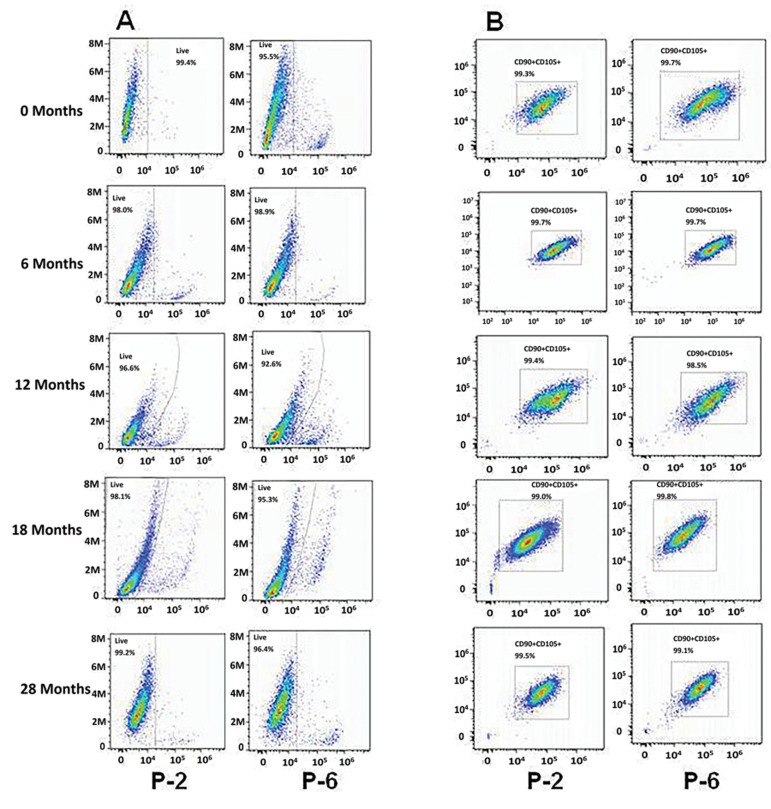
Representative images for flow cytometry studies across early and late passages, and at different time points. **A.** Cell viability remained unchanged, and **B. **Positive markers CD105 and CD90 were >95% in keeping with International Society for Cellular Therapy (ISCT) definitions for the mesenchymal stromal cells (MSCs).

Figure 3A and B are representative flow cytometry images of one lot for cell viability and positive markers, respectively, across early and late passages at different time points. This data indicated the stringency of the cell manufacturing processes whereby cell specific markers and viabilities were unaffected, and demonstrated that extended cryopreservation did not negatively impact the quality of the cells.

**Fig.4:**
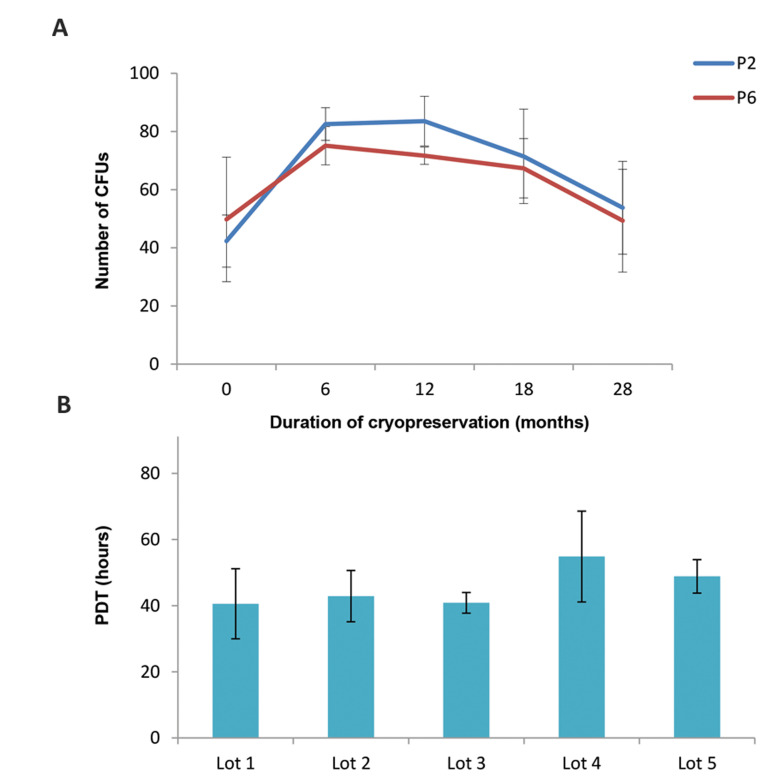
Colony forming unit (CFU) assay and population doubling time (PDT) were evaluated. **A.** The mean CFU potential at early and late passages, and at different time points (data for three different lots; two-way ANOVA, P>0.05). The stemness of the cells was retained despite cryopreservation. **B. **Depicts the mean PDT across five lots, at early and late passages and different time points, which remained largely unchanged except for the anticipated lot to lot variability.

Evaluation of the mean colony forming unit (CFU) potential revealed that the duration of cryopreservation and passage number had no effect on the number of colonies formed (Fig .4A). Although there was some lot-to-lot variability, this was anticipated due to the biological nature of the tissue. The efficiency of the CFU remained stable despite the passage number and cryopreservation, with an average of 66 ± 18 colonies at P-2 and 62 ± 12 colonies at P-6, and represents the quality and stemness of the MSCs.

The PDT indicates the age of the cells and maybe a better measurement of cell health than the passage number. The mean PDT was 45.01 ± 9.44 hours across all lots, passages and time points (Fig .4B). 

**Fig.5:**
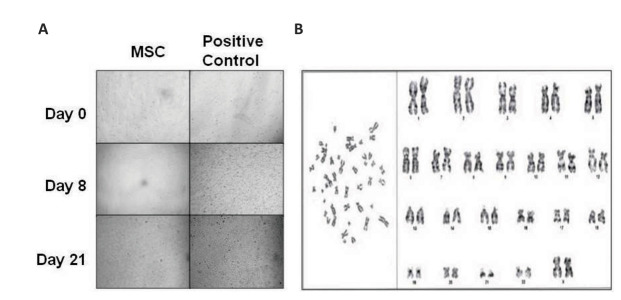
The soft agar assay was carried out to evaluate *in vitro* tumourigenicity. **A.** *In vitro* soft agar assay showed that after long-term culture and cryopreservation, the Wharton’s jelly mesenchymal stromal cells (WJ-MSCs) did not exhibit altered growth characteristics of plastic adherence and could not grow in soft agar. However, the positive control, the MCF7 breast cancer cell line, showed vigorous growth in soft agar and was not anchorage dependent, and ** B.** Representative karyotype (46, XX) of WJ-MSCs. Long-term culture and cryopreservation did not affect the karyotype.

After prolonged cell culture expansion and cryopreservation, the MSCs did not show any growth during the soft agar assay while the positive control clearly showed *in vitro* tumorigenic potential (Fig .5A).

Karyotype analysis revealed normal karyotype for all cells irrespective of lots, passages or duration of cryopreservation (Fig .5B).

Both the soft agar and karyotype data were important as cryopreservation, culture methods or reagents may contribute to genotypic instability; however, we did not see any evidence of the same in our studies.

**Fig.6:**
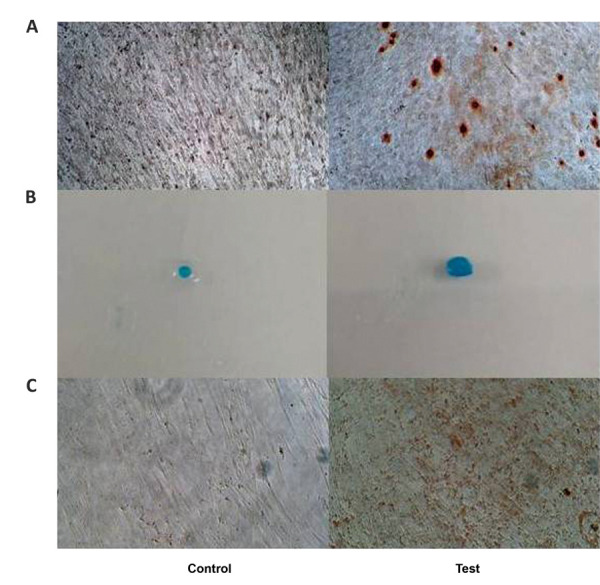
Representative images of differentiation studies of Wharton’s jelly mesenchymal stromal cells (WJ-MSCs). **A.** Osteogenic, **B.** Chondrogenic, and **C. **Adipogenic lineages.

The MSCs from all lots could be successfully differentiated into osteogenic, chondrogenic and adipogenic lineages at all passages and time-points post-cryopreservation (Fig .6A-C). The differentiation potential did not seem to vary. Since this was a qualitative analysis, quantification could not be carried out.

## Discussion

Our study established the stability of cryopreserved WJderived MSCs and demonstrated the feasibility of largescale working cell banks for clinical applications. The cells were well within the characterisation parameters that are globally recognized and accepted. Cell morphology, CD markers and differentiation capacities were consistent among different lots, passages and after 28 months of cryopreservation in liquid nitrogen. Moreover, there was no indication of chromosomal abnormalities in the karyotype studies, nor did the cells display any tumourigenic properties as evidenced in the soft agar assay. CFU studies revealed retained stemness and PDT showed largely unchanged growth patterns. 

Other groups have carried out stability studies of MSCs from alternate sources like bone marrow (26) and recommended the use of bone marrow MSCs up to passage 4 after which signs of differentiation were observed. Stultz et al. (27) applied spectral karyotyping as a tool to study bone marrow MSCs and used FBS as a growth supplement to culture cells up to passage 7. Their studies indicated early passage abnormalities which decreased inversely with increasing passages. Blázquez-Prunera et al. (28) utilised a commercially available human plasma fraction to develop xeno-free MSCs from adipose tissue, bone marrow and umbilical cord, and demonstrated the suitability of human plasma derived growth supplements for large scale expansions of stable cell populations. Patrikoski et al. (29) comparatively evaluated culture conditions of adipose derived MSCs using FBS, human serum and commercially available serum-free medium (STEMPRO). Although the results were comparable, they advocated chemically defined media in lieu of serum based media for better safety profiles during clinical applications. However, in our opinion, this greatly increases the cost of the final cell-based products. Many have reported the potential of human umbilical cord blood or cord tissue MSCs as the future of regenerative medicine due to numerous desirable traits that included availability, non-invasiveness, low immunogenicity and better proliferative and differentiation capacities (30, 31). There are emerging reports about translational work that used these cells for various applications (32-35). We have demonstrated that WJ-MSCs have the stability and requisite traits which are relevant for cell-based therapies.

Among other requirements, development of clinical grade cells for therapeutic use necessitates stability studies, especially if the intention is to create a large scale stem cell bank that involves expansion of cultured cells. This is required, not only for regulatory submissions, but to determine the shelf life of the cell-based product. We expanded the MSCs up to passage 6 without loss of critical parameters. These cells are normal diploid cells that follow the Hayflick limit (36) and can thus safely be used for cell-based therapies. 

## Conclusion

We were able to develop and study fully characterized WJ-derived MSCs of the master and working cell banks, both before and after cryopreservation. These studies were carried out in real time and demonstrate the stability, stemness and regenerative potential of the cell populations across various lots, early and late passages, and at different thawing time points without the loss of desirable characteristics up to 28 months. This validates the robustness of the cell expansion to establish a large scale cell bank that can be used for clinical applications. The risk of transplantation of these xeno-free MSCs is as much as or less than a blood transfusion and acceptable in terms of safety, and scientific and regulatory perspectives. Accordingly, we developed a method to derive human serum from plasma which, in addition to complement inactivation, was also subjected to cold viral inactivation and served as a safe, feasible and economical cell culture growth supplement. Further, we used recombinant trypsin in the disaggregation process and thus eliminated all possible sources of potential xeno contamination, which is in keeping with the global alignment towards xeno-free products. Establishment of a large scale cell bank involves various processes and multi-level validations that include procuring the umbilical cords, developing a master cell bank and finally establishing release criteria for the final cell-based product. We recommend that future studies evaluate the immune functions of MSCs; however, this study was a major step towards our goal of developing cell-based clinical applications. 
